# Effects of substratum and depth on benthic harmful dinoflagellate assemblages

**DOI:** 10.1038/s41598-020-68136-6

**Published:** 2020-07-09

**Authors:** Li Keat Lee, Zhen Fei Lim, Haifeng Gu, Leo Lai Chan, R. Wayne Litaker, Patricia A. Tester, Chui Pin Leaw, Po Teen Lim

**Affiliations:** 1 Bachok Marine Research Station, Institute of Ocean and Earth Sciences, University of Malaya, 16310 Bachok, Kelantan Malaysia; 2 Third Institute of Oceanography, Ministry of Natural Resources, Xiamen, 361005 China; 3 State Key Laboratory in Marine Pollution, Department of Biomedical Sciences, City University of Hong Kong, Hong Kong, 999077 China; 4 CSS Inc. Under Contract to National Oceanic and Atmospheric Administration (NOAA), Beaufort Laboratory, National Centers for Coastal Ocean Science, National Ocean Service, NOAA, 101 Pivers Island Road, Beaufort, NC 28516 USA; 5 Ocean Tester LLC, 295 Dills Point Road, Beaufort, NC 28516 USA

**Keywords:** Environmental sciences, Ocean sciences, Marine biology, Ecology, Ecosystem ecology

## Abstract

Microhabitats influence the distribution and abundance of benthic harmful dinoflagellate (BHAB) species. Currently, much of the information on the relationships between BHABs and microhabitat preferences is based on non-quantitative anecdotal observations, many of which are contradictory. The goal of this study was to better quantify BHAB and microhabitat relationships using a statistically rigorous approach. Between April 2016 to May 2017, a total of 243 artificial substrate samplers were deployed at five locations in the Perhentian Islands, Malaysia while simultaneous photo-quadrat surveys were performed to characterize the benthic substrates present at each sampling site. The screen samplers were retrieved 24 h later and the abundances of five BHAB genera, *Gambierdiscus*, *Ostreopsis*, *Coolia*, *Amphidinium*, and *Prorocentrum* were determined. Substrate data were then analyzed using a Bray–Curtis dissimilarity matrix to statistically identify distinct microhabitat types. Although BHABs were associated with a variety of biotic and abiotic substrates, the results of this study demonstrated differing degrees of microhabitat preference. Analysis of the survey results using canonical correspondence analysis explained 70.5% (horizontal first axis) and 21.6% (vertical second axis) of the constrained variation in the distribution of various genera among microhabitat types. *Prorocentrum* and *Coolia* appear to have the greatest range being broadly distributed among a wide variety of microhabitats. *Amphidinium* was always found in low abundances and was widely distributed among microhabitats dominated by hard coral, turf algae, sand and silt, and fleshy algae and reached the highest abundances there. *Gambierdiscus* and *Ostreopsis* had more restricted distributions. *Gambierdiscus* were found preferentially associated with turf algae, hard coral and, to a lesser extent, fleshy macroalgae microhabitats. *Ostreopsis*, almost always more abundant than *Gambierdiscus*, preferred the same microhabitats as *Gambierdiscus* and were found in microbial mats as well. With similar habitat preferences *Ostreopsis* may serve as an indicator organism for the presence of *Gambierdiscus*. This study provides insight into how BHAB-specific microhabitat preferences can affect toxicity risks.

## Introduction

Benthic harmful algal blooms are important due to their potential health and ecological impacts, as well as their detrimental effects on commercial fisheries and tourism^1^. Many species of benthic dinoflagellates in the genera *Gambierdiscus*, *Ostreopsis*, *Fukuyoa*, *Prorocentrum*, *Coolia* and *Amphidinium* have been implicated in production of a diverse array of bioactive compounds that impact human health and disrupt marine ecosystems. Most notably, *Gambierdiscus* produce ciguatoxins that bioaccumulate in marine food webs^2–5^. Consuming fish or shellfish contaminated with ciguatoxins results in ciguatera poisoning (CP)^1,6–9^ and symptoms of intoxication range from mild gastrointestinal or neurological disturbances to several prolonged illnesses or death^10^. CP is the most studied of the BHAB caused illnesses and is a recognized health threat throughout most tropical regions^11,12^. Certain *Ostreopsis* species produce palytoxin-like compounds and analogues^13–15^ that have been related (although not completely proven) to human respiratory irritation by inhalation or dermatitis by cutaneous contact^16,17^. These toxins can cause clupeotoxicity^18,19^ and palytoxicosis^17,20,21^ as well. The adverse effects of toxic *Ostreopsis* to marine organisms have been well documented (e.g., crustaceans^22,23^; juvenile fish^23^; polychaetes^24^; sea urchin^25,26^; and bivalves^27^) along with their ability to cause massive benthic ecosystem disruptions^28–30^. Several benthic *Prorocentrum* species produce okadaic acid and dinophysistoxins^31–38^ implicated in causing diarrheic shellfish poisonings^39–41^. Bioactive compounds identified from *Coolia* and *Amphidinium* are known to negatively affect marine life^42–48^, though no associated human intoxications have been reported.

BHABs inhabit a wide range of marine habitats and have close associations with biotic and abiotic bottom substrates including algal turf, macrophytes, seagrasses, corals, denuded coral rubble, rocks and sediment^2, 49–53^. These substrates, in combination with abiotic factors such as temperature, salinity and light form microhabitats that influence the relative distribution and abundance of BHABs. The degree to which certain species are favoured will determine the types and amounts of toxins entering the marine food chain. How factors such as light, temperature and salinity affect BHAB species, particularly *Gambierdiscus*, have been actively investigated^54–60^. In contrast, the precise ways microhabitats influence species distribution and abundance remains one of the least studied areas of BHAB ecology^53,61–64^. The potential importance of microhabitats is illustrated by studies on increased incidences of CP illnesses after large scale reef disturbances by hurricanes or dredging for an airport construction site that caused a shift from coral to macroalgal/turf algae dominated microhabitats^11,65–69^.

In a proof of concept study, Yong et al.^63^ quantified the importance of microhabitats in influencing BHAB composition and abundances. They used a standardized sampling method^70–72^ combined with digital underwater imagery to quantify various bottom substrates^73^. In this expanded follow-on study, sampling was done at the original coral reef ecosystem near Pulau Rawa, Malaysia^63^ and expanded to include four additional sites in the same region. The roles of temperature and depth in structuring microhabitats and associated BHAB composition and abundances were examined as well.

## Methods

### Study sites

**Figure 1:**
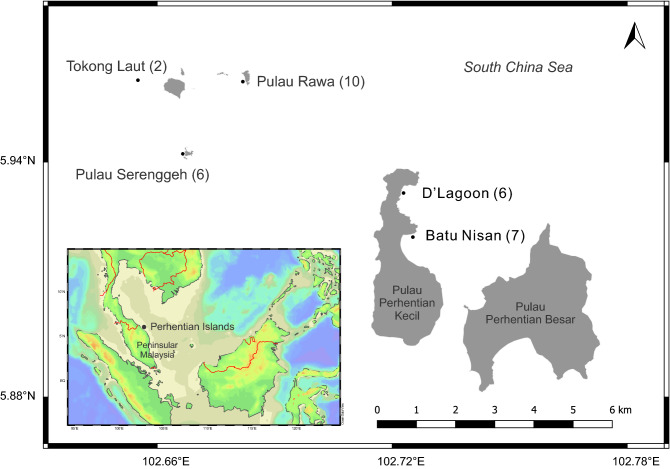
Map of Perhentian Islands Marine Park off the coast of Peninsular Malaysia, with the sampling sites: Pulau Rawa, Pulau Serenggeh, Tokong Laut, D’Lagoon, and Batu Nisan. The number of sampling efforts at each site throughout the sampling period is given in parentheses. The maps were generated by using Ocean Data View v. 5.3.0 (https://odv.awi.de) and QGIS v. 3.12.3 (https://qgis.org).

Sampling was undertaken at Perhentian Islands, Terengganu, Malaysia by SCUBA diving between April 2016 and May 2017. The specific sites examined were chosen to represent typical benthic habitats in the region. These sites included Pulau Rawa (5° 57′ 41.28″ N, 102° 40′ 57.25″ E), Pulau Serenggeh (5° 56′ 30.99″ N, 102° 40′ 3.46″ E), Tokong Laut (5° 57′ 39.49″ N, 102° 39′ 18.26″ E), D’Lagoon (5° 55′ 42.34″ N, 102° 43′ 26.78″ E) and Batu Nisan (5° 55′ 16.19″ N, 102° 43′ 40.50″ E) (Fig. 1). The Pulau Rawa and Pulau Serenggeh sampling sites are located on uninhabited islands. Both sites encompassed sheltered, shallow reef flats and gradually sloping from 5 to ~ 20 m. Tokong Laut is a relatively deep pinnacle, dominated by sandy/silty substrate and high currents. It was the deepest site sampled ranging from ~ 12 to ~ 25 m. The D’Lagoon and Batu Nisan sites are located at Perhentian Kecil Island. D’Lagoon is a relatively sheltered, low complexity fringing reef while the Batu Nissan site is a relatively exposed, higher complexity fringing reef.

### Physical data collection

The depths at each sampling location where screens were deployed were determined using a dive computer. Data sets for seawater temperatures and light intensity were obtained using a HOBO Pendant temperature/light 64 k data logger (Onset Computer Corporation, MA, USA) at 3 m at Pulau Rawa. A logger also was deployed at 10 m at the same site for part of the study to measure how daily temperature varied with depth. Only the maximum daily water temperatures observed at the 3 and 10 m depths were plotted. In addition, average maximum daily temperatures were calculated for the inter-monsoon, southwest monsoon and northeast monsoon seasons and included as part of the temperature time series graph.

Both light sensors deployed at 3 and 10 m malfunctioned so the time series for light was lost. As an alternative means of approximating the light versus depth relationship, functioning light loggers were deployed at 3, 6, 10, 15 and 18 m at Pulau Rawa and the relationship between photon flux density (PFD, µmol photons m^−2^ s^−1^) versus depth was determined as detailed in Supplementary Data 1. For subsequent analyses, the PFD versus depth relationship was used to estimate approximate light levels at each sampling point. Because of the way this relationship was determined, the PFD measurements should be regarded as an interchangeable proxy for depth as well as an approximate measure of light availability.

### Sample collection, processing and microhabitat mapping and classification

An artificial substrate sampling method utilizing fiberglass window screen mesh^63,70^ was employed in this study. The screens were deployed by SCUBA, retrieved 24 h later by carefully placing the screen in a wide-mouth 1-l bottle underwater (Supplementary Data 2). In the laboratory, the screens were shaken vigorously for 5–10 s to dislodge the attached cells. Samples were passed through a 200 µm sieve to remove detritus or particles. The filtrates were then filtered onto a 0.2-μm nylon membrane filter. The membrane filter was transferred into a 50-ml tube, filled with 30 ml of filtered seawater, and preserved with 1% acidic Lugol’s iodine solution for cell enumeration.

Cell abundances of five groups of benthic harmful dinoflagellates: *Amphidinium*, *Coolia*, *Gambierdiscus*, *Ostreopsis*, and *Prorocentrum* were enumerated (3–5 replicate counts) using a Sedgewick Rafter counting chamber under a Leica DM750 microscope (Leica, Germany) at 200 × magnification. Cell abundance was expressed as cells 100 cm^−2^ as in Tester et al.^70^.

To characterize the benthic dinoflagellate assemblages in relation to the microhabitat variability, the bottom substratum where the screens were deployed, were characterized simultaneously using a photo-quadrat method. This method utilized a waterproof digital camera mounted perpendicularly 1 m above a 0.25 m^2^ quadrat. This assemblage was used to photograph the bottom substratum each time a sample was taken (Supplementary Data 2). Digital underwater images were then analyzed for percent coverage of various bottom substrates using CoralNet^73^ (https://coralnet.ucsd.edu). The images were annotated with a total of 100 uniform annotation points based on general benthic reef community characterizations that were classified into nine benthic substratum types: invertebrates (Invt); coarse rubble and rocks (Rub); soft corals (SC); hard corals (HC); sponges (Spg); turf algal assemblages (Turf); upright fleshy macroalgae (Fles); fine sand and silt (Sd); microbial mats (MM) (Supplementary Data 2). All photo-quadrat images and annotation data are publicly available via CoralNet (https://coralnet.ucsd.edu/source/503/).

### Statistical analysis and data visualization

The data were first analyzed for normality with the Shapiro–Wilk test using PAST 3.25^74^. As the data were not normally distributed, a non-parametric one-way ANOVA on a Kruskal–Wallis rank with a Dunn’s multiple comparison test was used to test for significant differences between benthic harmful dinoflagellate assemblages and locality or microhabitat clusters. The distribution of benthic harmful dinoflagellates at each sampling point, in different benthic microhabitats and depths were conceptualized through bubble plots using *ggplot2*^75^. To evaluate the degree of benthic microhabitat heterogeneity, a cluster analysis with a Bray–Curtis dissimilarity matrix was performed based on the benthic substrate percent coverage; a dendrogram was constructed by *vegan*^76^ in R (R Core Team^77^). Non-metric multidimensional scaling (*n*MDS) was used to visualize the correspondence between distinct major clusters of benthic substrates (Supplementary Data 3). One-way analysis of similarity (ANOSIM^78^) was performed to test significant differences between the benthic microhabitat clusters. SIMPER analysis was used to assess the average percent contribution of microhabitat characteristics towards dissimilarity between clusters formed in *n*MDS and to identify probable major contributors of the differences detected in ANOSIM (Supplementary Data 3). These analyses objectively identified distinct microhabitat types based on the various substrates present.

A heatmap, where different color intensity represented the percent cover of each substrate type at each quadrate sampled over the course of the study was generated using *Heatplus*^79^. The heatmap was arranged so samples from different sites falling into each of the microhabitat types were plottted together. This convention made it easy to visualize which of the different substrate types (HC, Invt, SC, Spg, MM, Sd, Rub, Fles, or Turf) defined each microhabitat type. Next, the percent contribution of each of the five genera of benthic dinoflagellates to the total assemblage was determined by dividing the number of cells belonging to each genera by the total number of cells contributed by all five genera in a quadrat and multiplying by 100. These data were plotted in the same order within habitat type as used in plotting the substrate heatmap. Plotting the samples in the same order for the microhabitat cluster analysis, the heatmap of substrate type and the generic-specific heatmap allowed a direct comparison of habitat types, substrate types and the relative abundance of the different BHAB genera.

Because not all microhabitat types were distributed equally among sampling sites, the proportional distributions of each microhabitat type at each study site were calculated (in percentage) and presented as a stacked bar chart. It is important to note how the different substrate types were distributed with depth to determine the extent to which depth preferences by any genera were due to a factor such as light or temperature versus unequal distribution of substrate types with depth. To accomplish this, the distributions of the nine benthic substratum types as functions of depth were presented as a violin plot using *ggplots*.

Published data on the relative cell counts from screen sampling devices for *Amphidinium*, *Coolia*, *Gambierdiscus*, *Ostreopsis*, and *Prorocentrum* from various field studies were collated for comparative purposes (Supplementary Data 4). The habitat types and sample locations from each study were included. The goal was to determine if consistent patterns in relative abundance among the different genera measured using the screen method emerged when sites from different geographic locations, including those from this study, were compared.

To illustrate the distributions of the genera at the different depths, the abundance of each genus for each quadrat sample were plotted as a function of habitat type on the x-axis and depth on the y-axis. The abundances were indicated by different sized circles and the circles were colour coded to identify the sampling location. Canonical correspondence analysis (CCA) was used to infer the underlying relationship between the benthic harmful dinoflagellate assemblages and benthic substrate characteristics, depths, and irradiances. CCA is a constrained multivariate ordination technique that extracts major gradients among combinations of explanatory variables in a dataset and requires samples to be both random and independent. Data for cell abundances were Hellinger-transformed prior to CCA to ensure the data met the statistical assumptions of normality and linearity. The analysis was performed using *vegan*. The significance of variation in benthic harmful dinoflagellates assemblages explained by the explanatory variables was tested using an ANOVA-like Monte Carlo permutation test as implemented in *vegan*.

## Results

### Sampling frequency

**Figure 2:**
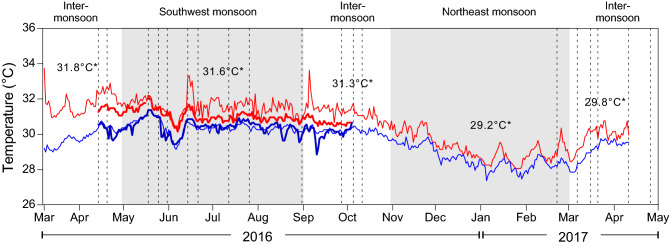
Seawater temperature recorded at the depths of 3 m (thin lines) and 10 m (thicker lines) from March 2016 to April 2017. Red lines represent daily maximum temperatures, blue lines represent daily minimum temperatures; * indicates average temperatures in the dry, wet and inter-monsoons. Dash lines indicate when samples were collected.

A total of 234 screens were deployed for 24-h periods and collected from various microhabitats and depths between 1 and 25 m at five different locations between April 2016 and May 2017. Sampling dates are indicated by the vertical lines in Fig. 2. The number of sampling sites at each location are provided in Table 1.

**Table 1:** Categories of benthic microhabitats of Perhentian Islands based on the benthic biological and physical substrates, the eight geomorphic zones were clustered based on percentage covers of substrates (see Fig. 3; Supplementary Data 2 and 3).

Cluster	Habitat description	Major substratum	Macroalgal coverage (%)	Screen (*n*)
1	> 40% of invertebrates (e.g., giant clams, sponges, corallimorpharians)	Invertebrates (Invt)	None—less (< 20%)	12
2	> 50% are coarse coral rubble and/or rocks	Rubble and/or rock (Rub)	None—less (< 30%)	15
3	> 50% of soft corals cover	Soft coral (SC)	None	3
4	50–100% covered by hard corals; some consisted of less than 30% of rubbles with turf algae	Hard coral (HC)	None—less (< 30%)	66
5	Micro-filamentous turf algal assemblages	Turf algae (Turf)	Abundant (> 50%)	64
6	Dominated by fleshy macroalgae (green/brown/red algae)	Fleshy Macroalgae (Fles)	Abundant (> 50%)	26
7	High coverage of sandy areas (> 60%)	Sand and silt (Sd)	None	38
8	High coverage (> 70%) of microbial mats comprised of cyanobacteria or diatoms that colonized sand or rubble substrates	Microbial mats (MM)	None	10

### Water temperature and light intensity

Over the course of the study, the maximum Perhentian Islands water temperatures at 3 m varied between a minimum of 28.1 °C and maximum of 33.7 °C (average of 30.7 °C; Fig. 2). Temperatures at the water depth of 10 m were more stable, ranging between a minimum of 30.2 °C and maximum of 32.2 °C (average of 31.0 °C; Fig. 2). Warmer temperatures occurred between April and September during the southwest monsoon period with lower temperatures observed between December–February during the northeast monsoon season. The depth of the photic zone in Perhentian Islands is estimated at ~ 23 m (Supplementary Data 1), with surface maximum light intensities of ~ 3,000 µmol photons m^−2^ s^−1^.

### Delineation of microhabitats

**Figure 3:**
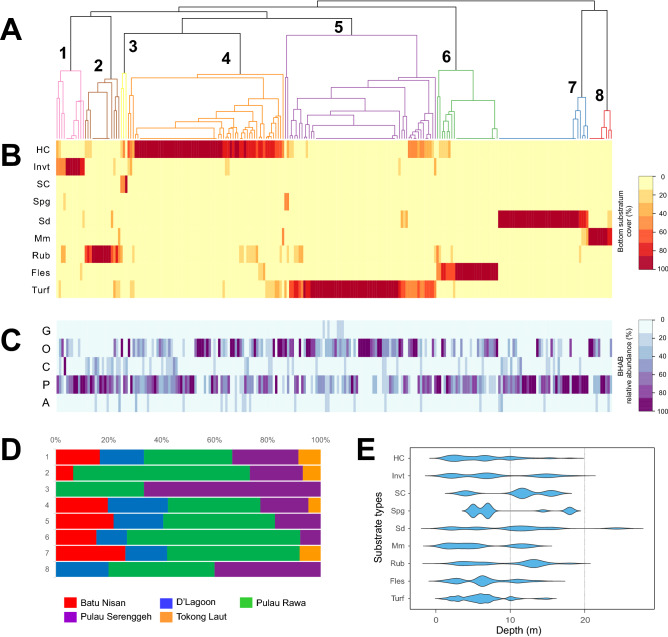
(**A**) Dendrogram revealed eight geomorphic zones of benthic microhabitats in Perhentian Islands where the screens deployed (*n* = 234). (**B**) Heatmap of benthic substratum % cover. (**C**) Heatmap showing the relative BHAB abundances. G, *Gambierdiscus*; O, *Ostreopsis*; C, *Coolia*; P, *Prorocentrum*; and A, *Amphidinium*. (**D**) A bar plot showing the relative proportion of microhabitat types at each site (in percentage). (**E**) Violin plot showing the depth distribution of various benthic substrates. *HC* hard corals, *Invt* invertebrates, *SC* soft corals, *Spg* sponges, *Sd* sand, *MM* microbial mats, *Rub* coarse rubble and rocks, *Fles* upright fleshy macroalgae, *Turf* turf algal assemblage.

The Bray–Curtis dissimilarity cluster analysis revealed the benthic substrates fell into eight different microhabitat types (Fig. 3A) with descriptions of the dominant features for each presented in Table 1. This microhabitat classification was further supported by the *n*MDS plot within a stress factor of 0.06 (Supplementary Data 3). Benthic microhabitats within the defined clusters were significantly different (ANOSIM, Global *R* = 0.9857, *p* < 0.0001). The results of the SIMPER test showed an overall average dissimilarity between the defined clusters (Supplementary Data 3^3^. The relative contribution of the substrate types for all the samples assigned to each microhabitat type are shown in the Fig. 3B heatmap. A total of eight microhabitat types were identified, each primarily defined by a particular, dominant substrate type. These were (1) Invt, (2) Rub, (3) SC, (4) HC, (5) Turf, (6) Fles, (7) Sd, and (8) MM. Note the benthic microhabitat dominated by microbial mats (microscopic algae such as diatoms and cyanobacteria) formed a distinct microhabitat different from that dominated by turf algae. The contribution of each BHAB genera, as % of total number of cells counted relative to the total number of all BHAB cell counted in the corresponding samples, is shown in Fig. 3C.

The eight distinct benthic microhabitats were patchily distributed across the five sampling sites (Fig. 3D). For example, microhabitat SC was only found on Pulau Rawa and Pulau Serenggeh (Fig. 3D). At the other extreme, HC microhabitat was found at each of the sampling sites and Turf microhabitat was found at all sites except Tokong Laut.

### Relative abundance and distribution of BHAB genera across habitats in the Perhentian Islands

**Figure 4:**
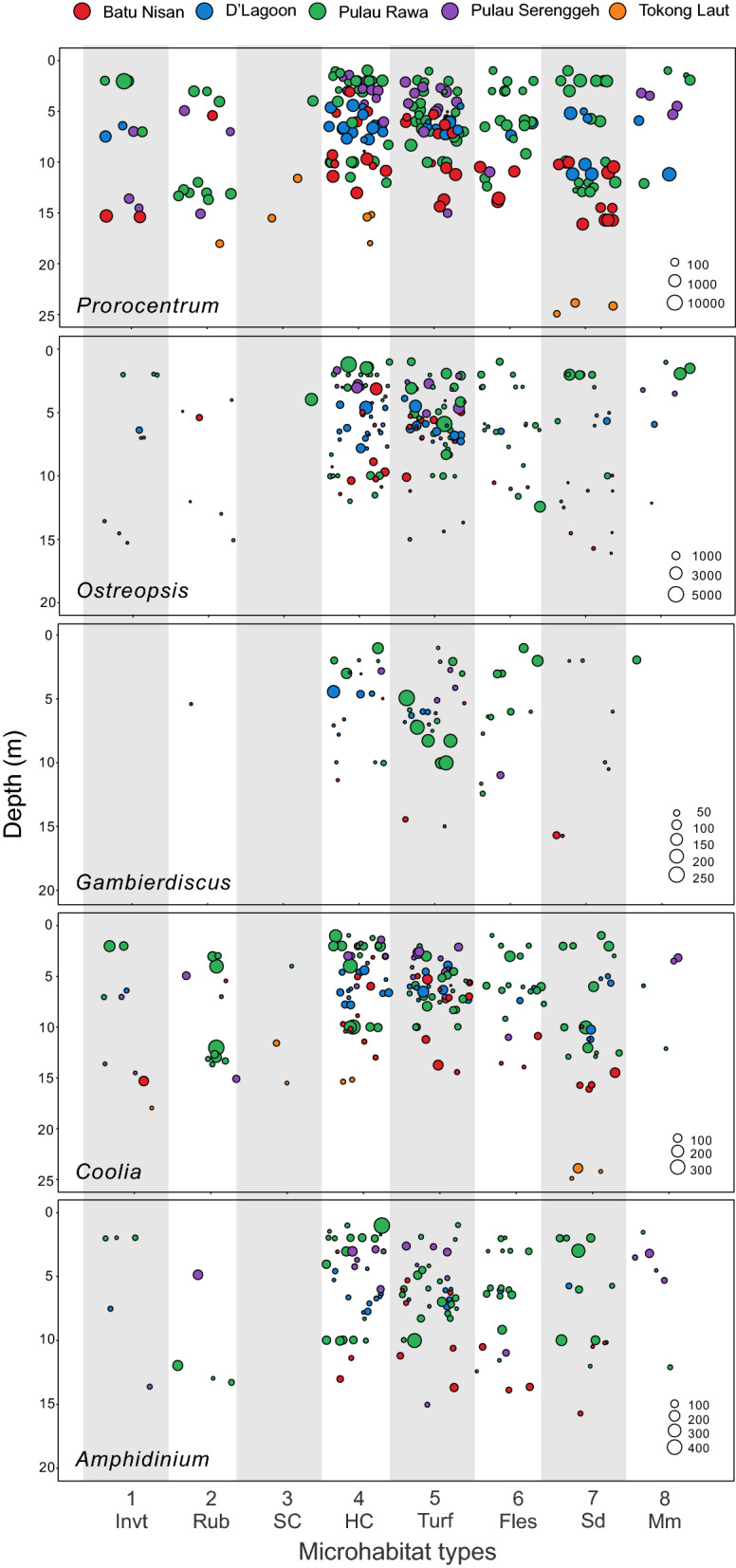
Abundances of benthic harmful dinoflagellates at various benthic microhabitat clusters and depth profile across the five sampling sites in Perhentian Islands, with respective size of circles representing cell abundances [cells 100 cm^−2^]. The axis lists the microhabitat clusters 1–8 determined from Fig. 3A and below those cluster numbers are the dominant habitat type found in each cluster. These are (1) Invertebrates (Invt); (2) coarse rubble and rocks (Rub); (3) soft corals (SC); (4) hard corals (HC); (5) turf algal assemblages (Turf); (6) upright fleshy macroalgae (Fles), (7) fine sand and silt (Sd); and (8) microbial mats (MM).

Overall, the benthic microhabitats HC and Turf supported the highest abundances of benthic harmful dinoflagellates as compared to other benthic microhabitat types (Fig. 4). Microhabitats Invt, Rub, SC, and MM supported lower BHAB abundances (Fig. 4).

Relative abundances of the five genera of benthic dinoflagellates differed over the microhabitats examined (Figs. 3, 4). Individual habitats were usually dominated by *Prorocentrum* (46–71% of total cells counted), except in Turf microhabitat (34%) where *Ostreopsis* was the most abundant group (51%). Consistent with its numerical dominance, the distribution of *Prorocentrum* (indicated by the percentage of screen samples in which at least one cell was observed in a habitat type divided by total number of screen samples collected in that habitat × 100) was homogenously distributed across microhabitat types being present in 99% of all screen samples counted (Kruskal–Wallis, *p* = 0.159; Fig. 4). This genus was most abundant in microhabitats HC, Turf, Fles, and Sd, though the absolute maximum *Prorocentrum* concentration (1.4 × 10^4^ cells 100 cm^−2^) was observed in a sample from the Invt microhabitat (Fig. 4).

*Ostreopsis* was the second most abundant genus observed comprising up to 51% of the total number of cells counted across all microhabitats. This genus was most abundant in HC (5.8 × 10^3^ cells 100 cm^−2^) and Turf microhabitats (5.6 × 10^3^ cells 100 cm^−2^), with relatively high occurrences among samples collected in these microhabitats (89.4 and 98.4%, respectively; Kruskal–Wallis, *p* < 0.0001). *Ostreopsis* also was frequently found in Fles microhabitat (occurrence = 88.5%, *n* = 26), but maximum abundance was lower (2.5 × 10^3^ cells 100 cm^−2^) than those found in HC and Turf microhabitats (Fig. 4). Lesser concentrations were also present in 60–75% of all samples counted from the Sd, MM, and Invt microhabitats. Occurrence in Rub and SC were even lower, 40% and 33%, respectively (Fig. 4).

*Gambierdiscus* achieved highest abundances in HC and Turf microhabitats (Fig. 4), with the maximum abundance (255 cells 100 cm^2^) found in Turf microhabitat (Fig. 4). Lower, but still relatively high cell concentrations were found in Fles microhabitat. Habitat specificity of *Gambierdiscus* is especially clear, as cells were not found in Invt or SC microhabitats and at low frequencies in samples from Rub, Sd, and MM microhabitats (7–29%) (Figs. 3C, 4).

*Coolia* did not show significant differences in distribution (Kruskal–Wallis, *p* = 0.176) in their frequency of occurrence among microhabitats (present in > 70% of samples in all habitat types, except the MM microhabitat with 40% occurrence). The highest cell abundance (368 cells 100 cm^2^) was observed in microhabitat 2 (Rub) with similarly high concentrations in HC, Turf, Fles, and Sd microhabitats (Fig. 4).

Although abundances of *Amphidinium* in various habitats were low (0–7.3% of total cells counted), the frequency of occurrences in some microhabitats was relatively high (27–81% of samples counted), except in the SC microhabitat where no cells were detected. The highest occurrence of *Amphidinium* in samples (81% of samples counted) was in the Fles microhabitat. Maximum abundances occurred in HC microhabitat with slightly lower maximum concentrations found in Turf and Sd microhabitats followed by lower concentrations in the Fles microhabitat. Even lower abundances were observed in the Rub, MM and Invt microhabitats (Fig. 4).

In terms of specific localities, Pulau Rawa (sheltered, shallow reef system) hosted the highest maximum abundances of benthic dinoflagellates (2 × 10^4^ cells 100 cm^−2^; Table 2). The assemblages in Batu Nisan (relatively exposed fringe reef) were dominated by *Prorocentrum* (57.4%) while Pulau Rawa (sheltered, shallow reef) and Pulau Serenggeh (sheltered, shallow reef) were dominated by *Ostreopsis* (51.4 and 60.4%). This is likely the result of the uneven distribution of habitat types among sampling locations (Fig. 3D). Only low abundances of *Prorocentrum* and *Coolia* (~ 260 cells 100 cm^−2^) were observed in Tokong Laut (turbulent, ~ 12–25 m deep, sandy and silt substrate dominated seamount).

**Table 2:** Relative abundances (%), maximum (Max), minimum (Min), mean cell abundances (unit: cells 100 cm^−2^), and coefficient of variation (CV) of benthic harmful dinoflagellates in Perhentian Islands between April 2016 and May 2017.

	Pulau Rawa (*n* = 105)	Pulau Serenggeh (*n* = 35)	Batu Nisan (*n* = 44)	D’Lagoon (*n* = 40)	Tokong Laut (*n* = 10)
*Gambierdiscus*					
%	2.2	1.7	0.8	1.1	0
Max	255	91	59	162	0
Min	0	0	0	0	0
Mean	26.2 ± 4.9 (50.6)	13.7 ± 3.9 (23.0)	7.0 ± 2.2 (14.7)	13.8 ± 4.8 (30.0)	0
CV	2.0	1.9	2.6	2.7	0
*Ostreopsis*					
%	51.4	60.4	35.0	49.3	0
Max	5,750	2,566	3,125	3,681	0
Min	0	0	0	0	0
Mean	613.9 ± 103.9 (1,064)	492.3 ± 131.5 (777.9)	322.0 ± 86.2 (571.5)	602.7 ± 129.4 (818.3)	0
CV	1.7	1.6	1.8	1.4	0
*Coolia*					
%	5.7	6.3	4.6	4.1	32.8
Max	368	180	142	177	131
Min	0	0	0	0	0
Mean	68.1 ± 7.2 (74.0)	51.5 ± 7.6 (44.9)	42.7 ± 6.1 (40.1)	49.6 ± 6.4 (40.5)	39.5 ± 11.7 (37.0)
CV	1.2	1.0	1.1	0.9	1.1
*Prorocentrum*					
%	36.1	26.7	57.4	43.6	67.2
Max	13,950	585	1733	2,679	131
Min	43	73	29	0	0
Mean	431.4 ± 131.7 (1,350)	217.5 ± 17.3 (102.4)	528.5 ± 63.5 (421.2)	532.9 ± 84.0 (531.3)	81.10 ± 12.8 (40.4)
CV	3.1	0.6	0.8	1.0	0.6
*Amphidinium*					
%	4.6	5.0	2.2	1.9	0
Max	456	158	113	105	0
Min	0	0	0	0	0
Mean	55.4 ± 7.1 (72.4)	40.7 ± 7.8 (46.2)	20.0 ± 4.6 (30.3)	22.7 ± 4.2 (26.9)	0
CV	1.5	1.3	1.7	1.5	0

Mean values are reported as mean ± standard error (standard deviation). CVs are calculated as standard deviation/mean. *n*, number of screens deployed and analysed.

Results from the literature survey indicated that BHAB assemblages were most often dominated or co-dominated by *Prorocentrum* and *Ostreopsis* (Supplementary Data 4). *Gambierdiscus* represented a minor portion of the assemblages present except for two samples taken during local blooms^70,81^. *Coolia* were not sampled as often so their patterns of abundance were not readily assessed. When *Coolia* concentrations were measured, data indicated they can numerically dominate benthic microalgal assemblages. *Amphidinium* was sampled less frequently still and were typically present only at low abundances relative to other species.

### Canonical correspondence analysis (CCA)

**Figure 5:**
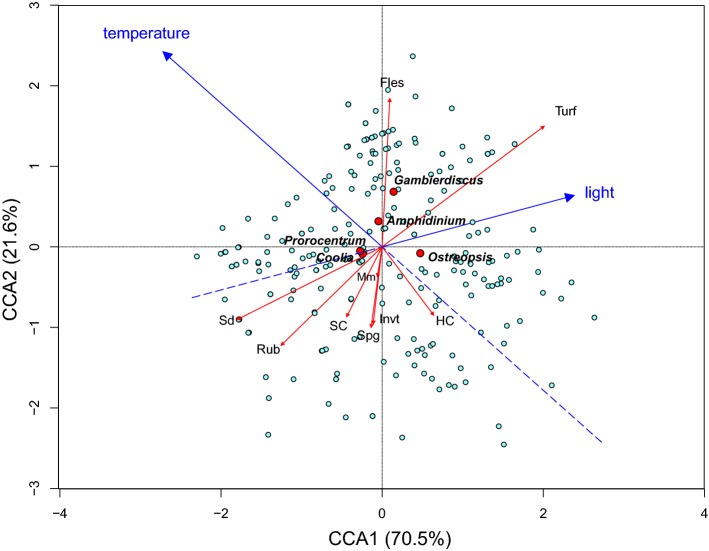
Canonical correspondence analysis (CCA) ordination of five BHAB groups elucidates their relationships with the benthic microhabitat characteristics, irradiance (surrogate for depth) and temperature.

The canonical correspondence analysis (CCA) was carried out to assess the degree to which the various BHAB genera were associated with different benthic substrate characteristics, light (as an inverse proxy for depth) and temperature (Fig. 5). The horizontal first axis (CCA1) explains 70.5% (eigenvalue, 0.1088, *p* = 0.001***) of this constrained variation, and the vertical second axis (CCA2) explains 21.6% (eigenvalue, 0.333, *p* = 0.001***). Taken together, both axes of the data set explained 92% of total inertia, which is highly significant at *p* = 0.001*** (Monte Carlo Permutation test, *n* = 999; F = 8.94), indicating strong correlations between the BHAB abundances, substrate types, light level and seawater temperature (Supplementary Data 5).

Turf algae had the greatest influence on CCA1 in a positive direction (F = 15.77, *p* = 0.001***), while Rub and Sd had the greatest influence in the negative direction (Rub, F = 4.57, *p* = 0.002**; Sd, F = 1.88, *p* = 0.112). Light further influenced CCA1 in a positive direction while temperature influenced CCA1 strongly in the negative direction (Fig. 5; Supplementary Data 5). These factors clearly separated *Gambierdiscus*–*Ostreopsis* (CCA1 > 0) and *Prorocentrum*–*Coolia* (CCA1 < 0). Along CCA2, which explains less than a quarter of the total variation, the factors having the most influence in the positive direction (CCA2 > 0) were temperature, fleshly macroalgae, turf algae and to a lesser degree light. Factors influencing CCA2 in the negative direction (CCA2 < 0) were HC and Rub. *Amphidinium* and *Gambierdiscus* were positively associated with temperature, compared to *Prorocentrum* and *Coolia* which were not strongly influenced by it. *Ostreopsis* was negatively correlated with temperature indicating abundances were higher during the cooler sampling periods.

*Prorocentrum* and *Coolia* were negatively correlated with light (surrogate for depth), but only marginally so. This slight negative relationship may have been influenced by these two genera being the only ones present at the deepest depths (Fig. 4). *Gambierdiscus*, *Amphidinum*, and *Ostreopsi*s were positively associated with light as compared to *Prorocentrum* and *Coolia*, which were negatively weighted, but only slightly. *Amphidinium*, *Gambierdiscus*, and *Ostreopsis* were positvely associated with Turf. *Gambierdisucs* and *Amphidinium* were assocated with Fles and *Ostreopsis* with HC (Fig. 5; Supplementary Data 5). *Prorocentrum* and *Coolia* were associated with Sd and Rub but not strongly. This is consistent with their wide occurrence across the various habitat types (Fig. 4).

### BHAB distribution with depth

With respect to depth, the BHAB assemblages were abundant at the depths of 1–10 m, with the average maximum abundances mostly observed at these depths (Fig. 4). For example, the maximum *Gambierdiscus* abundance was observed in a Turf microhabitat at 4.9 m. Similarly, maximum *Ostreopsis* abundances occurred in a HC microhabitat at 1.2 m, *Coolia* in a Rub microhabitat at 12 m, *Prorocentrum* in a corallimorph (Invt) dominated microhabitat at 7 m, and *Amphidinium* in a HC microhabitat at 1 m. *Prorocentrum* and *Coolia* were ubiquitous, occurring at all depths down to 25 m (Fig. 4). The results also revealed that both these genera were negatively associated with light (Fig. 5). In contrast, *Ostreopsis*, *Gambierdiscus* and *Amphidinium* are more aggregated at the depths of < 10 m. The greatest depth where *Ostreopsis*, *Gambierdiscus*, and *Amphidinium* were found was 16 m (Fig. 4). Interestingly, *Prorocentrum* and *Coolia* were the only genera observed at Tokong Laut and then only at depths > 12 m.

## Discussion

### Effect of benthic microhabitats on the BHAB assemblages

This study focused on expanding our understanding of the role microhabitat types play in controlling the distribution and abundance of benthic harmful algal bloom species (BHABs) in the genera *Amphidinium*, *Coolia*, *Gambierdiscus*, *Ostreopsis*, and *Prorocentrum*. Understanding how different microhabitats foster various BHAB genera is critical for understanding their relative contributions to toxin transfer in marine food webs, identifying hot spots or sentinel sites for monitoring and eventually, modelling efforts. Field efforts to examine the relationship between microhabitat and BHABs were hampered previously by lack of ways to objectively define habitat types and a standardized, uniform BHAB cell sampling method. The current investigation used systematic classification of habitat types in photographs from each sampling site in conjunction with Bray–Curtis dissimilarity cluster analysis to define different microhabitat types (Figs. 3, 4). Cell abundances of the BHAB genera were measured using a method that standardized sampling surface areas. This method enabled normalization of benthic dinoflagellate abundances to a known surface area for comparison among sites and studies^1,63,70–72,80,81^ despite the heterogeneity and complexity of benthic habitats^63^.

Most previous studies only sampled macrophyte hosts as the target substrate leading to many contradictory data regarding their association with various BHAB genera (as reviewed in Tester et al.^64^). Other potential hosts such as hard coral colonies, turf algal assemblages, as well as abiotic substrates like rubble, rocks and sand sediment were less frequently sampled. Field collection of macrophyte substrates in BHAB studies may seem convenient because they are easily accessible, however, in this study there was substantial variability of BHAB species occurrence and abundances in various benthic microhabitats, including the Fles habitat type. The high coefficient of variation (CV > 1.0; see Table 2, Supplementary Data 6) is indicative of significantly patchy distributions. The application of an artificial substrate sampling technique coupled with benthic photo-quadrat surveys confirmed other types of benthic substratum, besides macrophytes, support high BHAB abundances.

Although BHABs occupied most of the microhabitats examined in the Perhentian Islands, Malaysia, our results demonstrated that some BHAB genera exhibited a degree of preference towards specific microhabitats. *Prorocentrum* and *Coolia* were widely distributed among each of the habitat types indicating a broad ecological niche (Fig. 4). The CCA showed the two genera tightly clustered, consistent with their having similar microhabitat preferences (Figs. 4, 5). Both genera were most abundant in the hard coral and turf-dominated microhabitats, followed by sand and fine silt, fleshy macroalgae, invertebrates, course rubble and microbial mats (Fig. 4). Regarding occurrence, neither genus exhibited a strong habitat preference.

*Ostreopsis* showed a strong preference for microhabitats dominated by hard corals and turf algae where they reached their highest abundances but were also abundant in microhabitats dominated by fleshy macroalgae, sand and fine silt, invertebrates, microbial mats and course rubble and rock (Figs. 4, 5). The result of CCA indicated the genus was negatively associated with temperature but not light, suggesting higher abundance samples were collected when ambient temperatures were lower. The results also indicated that of the diverse microhabitats where *Ostreopsis* occurs, highest densities are most likely to be found in hard coral, consistent with the cell density estimates shown in Fig. 4. Overall, these observations support *Ostreopsis* species as opportunists capable of colonizing a wide variety of living and non-living benthic substrates^30,62,82–86^. The high abundances on turf algae versus relatively low abundances observed on course rubble and rock substrates have implications for research in the Mediterranean, especially where coral reefs are absent. There, *Ostreopsis* are often noted as being strongly associated with hard substrates, particularly rock and manmade structures^30^, other than the dense macroalgal mats or turfs^82,87–89^. This association may be the algal turfs associated with the hard substrates and represent an area of research that could yield important insights into the population dynamics of toxic *Ostreopsis* species.

*Gambierdiscus* exhibited the most restricted microhabitat range of the five genera surveyed. They were found predominantly where substrates were dominated by turf algae, hard coral and to a lesser extent fleshy macroalgae (Fig. 4). They were either absent, or present at only low concentrations, in the other microhabitats (Fig. 4). The genus was also positively associated with increasing light and temperature indicating a preference for conditions occurring at shallower depths (Figs. 4, 5). Of the eight habitats defined in this study, the CCA indicated turf algae in association with higher temperature and light would represent the microhabitat most likely to support the highest *Gambierdiscus* cell densities. This conclusion is consistent with other studies showing an affinity of *Gambierdiscus* for turf algae^63,90^. Several published arguments have been advanced for why turf-dominated microhabitat are preferable. First is, turf algae provide larger surface area for occupancy as compared to fleshy macrophytes^2,49,53,91^. Secondly, structural architecture of turfs with spatial complexity are more likely to create a microhabitat with low micro-scale flow velocity that provides refugia against flow-related disturbances^53,92^. These results also argue against sampling only macroalgae as a means of estimating overall BHAB cell abundances because they are not the preferred microhabitat for BHAB species, particularly *Gambierdiscus*, the most toxic of the genera.

The habitat preferences of *Gambierdiscus* most closely resembled those of *Ostreopsis*. A major distinction was that *Ostreopsis,* is more broadly distributed among habitats. The highest *Gambierdiscus* cell concentrations were associated with turf algae microhabitat followed by the hard coral microhabitat, whereas the relative abundances in these two habitats were reversed for *Ostreopsis* (Fig. 4). The similarity in microhabitat preferences also suggests *Ostreopsis*, which are more abundant than *Gambierdiscus*, may serve as a good indicator of where *Gambierdiscus* are present (Fig. 4; Supplementary Data 4^64^).

*Amphidinium* was widely distributed among habitat types with greatest abundances observed in turf algae, fleshy macroalgae, hard coral, and sand and fine silt microhabitats (Fig. 4). Its abundances were lower in the other habitats and it was absent from all soft coral samples. CCA indicated that *Amphidinium* species were positively associated with higher temperature and light, likely to reach maximum densities in microhabitats with substrates dominated by algal turfs or fleshy macroalgae. This microhabitat distribution was similar to the broad range of microhabitats preferred by *Prorocentrum*. Again, whether this utilization of diverse microhabitats was due to only a few species with broad ecological niches, a larger number of species with specialized requirements or a combination of the two is unknown. A molecular survey of different microhabitats using genus specific rDNA primers and high throughput sequencing represents a promising means of addressing this question.

*Prorocentrum* were the numerically dominant species (Table 2) followed by *Ostreopsis*, *Amphidinium*, *Coolia*, and *Gambierdiscus*. The latter three genera, even when combined represent only a minor component of the overall BHAB assemblages on a per cell basis. The literature survey on studies using artificial substrate sampling methods also revealed *Prorocentrum* and *Ostreopsis* as the dominated or co-dominate genera at most locations (Supplementary Data 4). The only instance where *Gambierdiscus* dominated was during local blooms as observed in Belize^70^ and Canary Islands^81^. Otherwise, it formed only a minor component of the assemblage as observed in the current study. *Amphidinium* has been irregularly sampled making it more difficult to draw any conclusions about this genus. Where data do exist, *Amphidinium* represents a minor component of BHAB assemblages. In contrast to this study, the literature survey indicated *Coolia* abundances were sometimes numerically dominant, or co-dominant in habitats characterized by mixed macroalgae, rocks and sand (Fernández-Zabala et al.^81^; Supplementary Data 4).

### Depth distribution of BHAB assemblages

The depth distribution of BHAB species often yields contradictory information (reviewed in Tester et al.^64^). Some studies show increased abundances in the first few meters, others at 10–40 m depth and others show no difference with depth. The use of artificial substrates deployed at different depths provides an unbiased means of how the various BHAB genera are distributed with depth, including potential differences in light, temperature (Fig. 2), microhabitat type (Fig. 3) and wave action. Results from this study showed maximum abundances of *Gambierdiscus*, *Ostreopsis*, and *Amphidinium* occurred at depths < 10 m (Fig. 4). All three genera were positively associated with light in the CCA consistent with shallower depth distributions (Fig. 5). These observed distributions may be due to the availability of the preferred microhabitats, in this case, the warm-water coral, turf algae and macrophyte-dominated habitats (Fig. 3E) that were distributed preferentially toward the shallower waters. As shown in this study, Pulau Rawa which sheltered and encompassed the highest complexity of microhabitats as compared to other sites (Fig. 3D), hosted the highest abundances of all five BHAB groups among the sites studied (Table 2). The combination of relatively lower turbulent environments and greater microhabitat availability, particularly those including turf algae and hard corals, probably contributed the higher cell abundances observed. Many ecological studies also report increased species richness and abundance in more complex habitats^93,94^.

Conversely, *Prorocentrum* and *Coolia* exhibited a broader depth distribution and can be found in deeper waters (to ~ 25 m), with preferred microhabitat types distributed over all depths (Fig. 3E). *Prorocentrum* and *Coolia* were also the only two BHAB genera found in the deeper habitats at Tokong Laut, which is a high-energy pinnacle reef affected by stronger underwater currents and dominated by sand and silt (Fig. 3D). Although the effect of physical disturbance on habitat type was not directly studied, species belonging to these BHAB genera can be found in habitats with a moderately high level of turbulence^53,82^. They may benefit from small-scale turbulence in low nutrient habitats, which would increase nutrient diffusion rates and efficiency of cell nutrient uptake^95^. In the suite of species present, some may exhibit higher growth rates than those in other genera (i.e., *Gambierdiscus*), allowing them to better survive population losses due to turbulent dispersion such as that caused by the relative high current regime found at Tokong Laut. These genera were also proportionately more abundant on the sand and silty substrate predominating at this deeper site indicating they were adapted to utilize this substrate. However, relatively low sampling efforts at this site could have contributed to these findings. The association with the prevailing substrate type found at the deeper Tokong Laut site may also account for the slight negative association of these species with light.

The greater depth range exhibited by these two genera is not likely due to a greater capacity to cope with lower light levels. Dinoflagellates, in general, are low light adapted, most achieving maximal growth rates at 50–100 µmol photons m^−2^ s^−1^ compared to surface irradiance, often > 2,500 µmol photons m^−2^ s^−1^^64^. In clear waters, these irradiances can extend to 50 or 100 m. No systematic differences in photosynthetic capacity have been demonstrated among BHAB genera under light intensities less than 100 µmol photons m^−2^ s^−1^. Some species in each genus exhibit positive growth in light levels as low as 10–50 µmol photons m^−2^ s^−1^^54,57,59,60,64^. The greater problem for these species in shallower waters is photoinhibition. Benthic dinoflagellates cope with high light by taking advantage of shading by substrates and physiological methods such as variations in pigmentation^49,95–100^.

## Conclusion

The results of this study revealed that substrate variability in the microhabitats across depth-gradients determined the composition and differentially foster the abundance of BHAB species. This study and Yong et al.^63^ represent pioneering efforts to numerically evaluate the influence of benthic microhabitat heterogeneity on the abundance and distributions of BHABs. Both efforts provide a robust sampling and statistical analysis to classify the sites where BHABs were sampled based on the various benthic substrate types and allowed comparison of habitat diversity across all BHAB sampling sites. This approach can be used in designing monitoring programs at sentinel sites and provides insight into site specific differences in BHAB abundances and potential for toxins to enter marine food webs. It also seems clear that disturbances of the bottom substrates, such as coral reef degradation will markedly influence the BHAB assemblages^67–69^.

## Supplementary information

Supplementary file1 (DOCX 884 kb)

## Data Availability

All data generated during this study are included in this published article and its supplementary information files. The primary and secondary datasets are also available via figshare (https://figshare.com/projects/Effects_of_benthic_substratum_characteristics_and_depth_on_benthic_harmful_dinoflagellate_assemblages/81026).
