# Supplementary material for: Effects of substratum and depth on benthic harmful dinoflagellate assemblages
Source: Sci Rep. 2020 Jul 9;10:11251. doi: 10.1038/s41598-020-68136-6 (PMC7347539; doi:10.1038/s41598-020-68136-6)
Supplement: Supplementary file 1 — Supplementary file1 (DOCX 884 kb) [file 41598_2020_68136_MOESM1_ESM.docx]

**Supplementary Information**

**Effects of substratum and depth on benthic harmful dinoflagellate assemblages**

Li Keat Lee^1^, Zhen Fei Lim^1^, Haifeng Gu^2^, Leo Lai Chan^3^, R. Wayne Litaker^4^, Patricia A. Tester^5^, Chui Pin Leaw^1^*, Po Teen Lim^1^*

*^1^Bachok Marine Research Station, Institute of Ocean and Earth Sciences, University of Malaya, Bachok 16310 Kelantan, Malaysia*

*^2^Third Institute of Oceanography,* *Ministry of Natural Resources, Xiamen, 361005, China*

*^3^State Key Laboratory in Marine Pollution, Department of Biomedical Sciences, City University of Hong Kong, Hong Kong, 999077, China*

*^4^CSS Inc. under contract to National Oceanic and Atmospheric Administration, National Ocean Service, National Centers for Coastal Ocean Science, Beaufort Laboratory, 101 Pivers Island Road, Beaufort, NC 28516, USA*

*^5^Ocean Tester LLC, 295 Dills Point Road, Beaufort, NC 28516, USA*

Corresponding authors:

ptlim@um.edu.my (P.T. Lim), cpleaw@um.edu.my (C.P. Leaw)

**Supplementary Data 1:**

**Irradiance levels vs. depth determination**

The light sensor deployed as part of the long-term time series at Pulau Rawa. Therefore, to obtain an approximate estimate of the relationship between light and depth data loggers were deployed at 3, 6, 10, 15 and 18 m at Pulau Rawa for 24 h between 12-13 March 2019. The maximum daily irradiance measured in lux recorded at each depth. These daily maximal lux values were converted to photosynthetic photon flux density (PPFD) by a conversion factor of 0.0185 and plotted versus depth shown in panel A (Thimijan and Heins, 1982). The irradiance values were also log transformed and a light intensity versus depth regression determined: slope, -10.80 ± 0.7222 and y-intercept, 38.40 ± 1.979 (*R^2^* = 0.9824, *p* = 0.0001; panel B).

Reference:

Thimijan, R.W. & Heins, R.D. Photometric, radiometric, and quantum light units of measure: A review of procedures for interconversion. HortScience **18**, 818-822 (1982).

**Supplementary Data 2:**

1. **Microhabitat characterization by photo-quadrat method and artificial substrate sampling**

To characterize the benthic dinoflagellate assemblages in relation to microhabitat variability, the bottom substratum, where the screens were deployed, were characterized simultaneously using a photo-quadrat method (A, B). This method utilized a waterproof digital camera mounted perpendicularly to a 0.25 m^2^ quadrat (50 cm × 50 cm) to photograph the substratum from a distance of 1 m above the substrate (B). The artificial substrate consisted of a piece of fiberglass window screen of 10.2 cm × 15.2 cm (porosity of 1.0 mm) in size, attached to a fishing line with a weight and a sub-surface buoy (C), and suspended 20-cm above the substrate (Tester et al., 2014).

**A**

**B**

**C**

Reference:

Tester, P.A. et al. Sampling harmful benthic dinoflagellates: Comparison of artificial and natural substrate methods. Harmful Algae **39**, 8-25 (2014).

1. **Description of benthic substratum types**

Digital underwater images were analyzed for percent coverage of nine common benthic substrates using CoralNet (http://coralnet.ucsd.edu). The images were annotated with a total of 100 uniform annotation points based on general benthic reef community characterizations that were classified into nine benthic substratum types. All photo-quadrat images and the annotation data are publicly available via CoralNet (https://coralnet.ucsd.edu/source/503/).

| **Substratum types** | **Code** | **Biotic functional components** | **Possible taxa** |
| --- | --- | --- | --- |
| Invertebrates | Invt | Giant clams | *Tridacna maxima*, *T. squamosa* |
|  |  | Sea Anemone | *Heteractis* |
|  |  | Corallimorph | *Amplexidiscus*, *Discosoma* |
| Coarse rubble and Rock | Rub | - | - |
| Soft Coral | SC | Soft coral | *Sarcophyton*, *Melithaea*, *Sinularia* |
| Hard Coral | HC | Hard coral | *Acropora*, *Goniastrea*, *Porites*, *Pocillopora*, *Favia*, *Montastrea*, *Lobophyllia*, *Heliopora*, *Montipora*, *Turbinaria*, *Galaxea*, *Oulophyllia*, *Fungia*, *Astreopora*, *Hydnophora*, *Diploastrea*, *Echinopora*, *Gardineroseris*, *Platygyra*, *Euphyllia* |
| Sponges | Spg | Sponges | - |
| Upright and fleshy macroalgae | Fles | Rhodophyta | *Jania*, *Acanthophora* |
|  |  | Phaeophyta | *Lobophora*, *Dictyota*, *Padina*, *Dictyopteris*, *Turbinaria* |
|  |  | Chlorophyta | *Halimeda*, *Caulerpa* |
| Mixed assemblages of microfilamentous turf algae | Turf | Cyanophyta | *Lyngbya*, *Calothrix* |
|  |  | Rhodophyta | *Centroceras*, *Polysiphonia*, *Neosiphonia*, *Heterosiphonia* |
|  |  | Chlorophyta | *Cladophora* |
| Sand and silt | Sd | - | - |
| Mats-forming algae associated with cyanobacteria or diatoms | MM | - | - |

**Supplementary Data 3:**

1. **Grouping of benthic microhabitats - Non-parametric multidimensional scaling**

The dataset of microhabitat characteristics was analyzed using a non-parametric multidimensional scaling (*n*MDS) ordination to emphasize the correspondence between distinct major clusters representing diverse benthic microhabitat classes in a low-dimensional space (dimension = 3) with a stress factor of 0.06.

(1) Invertebrates (Invt); (2) coarse rubble and rocks (Rub); (3) soft corals (SC); (4) hard corals (HC); (5) turf algal assemblages (Turf); (6) upright fleshy macroalgae (Fles), (7) fine sand and silt (Sd); and (8) microbial mats (Mm).

1. **Grouping of benthic microhabitats – SIMPER test**

Pairwise comparisons between various benthic substratum clusters (SIMPER test) helped determine the overall average dissimilarity (%), the major substratum contributors and the percentage of contribution.

The results revealed Cluster 8 (microbial mats - Mm) exhibited the highest average dissimilarity when compared to Clusters 1 (Invertebrates - Invt), 2 (coarse rubble and rocks - Rub); 3 (soft corals - SC), 5 (turf algal assemblages - Turf) and 6 (upright fleshy macroalgae - Fles), with the major contributor to the dissimilarity being microbial mats. Cluster 7 (sand cover - Sd) showed the highest dissimilarity to Cluster 4 (HC), with sand being the major contributor to the dissimilarity. These two substratum characteristics were distinctive and did not share much similarity with other clusters. Clusters 5 (Turf) and 6 (Fles) have the highest average dissimilarity with fleshy macroalgae cover as the major contributor. The overlap Cluster 4 (HC) with four other clusters (Clusters 1 (Invt), 2 (Rub), 3 (SC), and 6 (Fles)) showed slight similarity in the benthic substratum characteristics.

| Cluster | Overall average dissimilarity (%) | Major contributor | Contribution (%) |
| --- | --- | --- | --- |
| 1 vs 2 | 87.93 | Invt | 43.09 |
| 1 vs 3 | 92.39 | Invt | 41.81 |
| 1 vs 4 | 90.32 | HC | 45.47 |
| 1 vs 5 | 94.16 | Turf | 43.19 |
| 1 vs 6 | 96.20 | Fles | 45.80 |
| 1 vs 7 | 97.60 | Sd | 48.40 |
| 1 vs 8 | 99.58 | Mm | 47.70 |
| 2 vs 3 | 81.86 | Rub | 40.88 |
| 2 vs 4 | 91.20 | HC | 45.93 |
| 2 vs 5 | 90.27 | Rub | 43.87 |
| 2 vs 6 | 94.11 | Fles | 47.03 |
| 2 vs 7 | 96.63 | Sd | 48.67 |
| 2 vs 8 | 97.66 | Mm | 47.61 |
| 3 vs 4 | 75.70 | HC | 43.45 |
| 3 vs 5 | 93.65 | Turf | 44.45 |
| 3 vs 6 | 95.06 | Fles | 47.01 |
| 3 vs 7 | 99.73 | Sd | 48.50 |
| 3 vs 8 | 100 | Mm | 47.50 |
| 4 vs 5 | 86.24 | Turf | 45.99 |
| 4 vs 6 | 92.12 | Fles | 47.67 |
| 4 vs 7 | 99.88 | Sd | 48.43 |
| 4 vs 8 | 99.30 | Mm | 47.48 |
| 5 vs 6 | 97.24 | Fles | 45.87 |
| 5 vs 7 | 98.44 | Sd | 48.38 |
| 5 vs 8 | 99.76 | Mm | 47.61 |
| 6 vs 7 | 99.93 | Sd | 48.40 |
| 6 vs 8 | 100 | Mm | 47.50 |
| 7 vs 8 | 92.26 | Mm | 50.00 |

**Supplementary Data 4:**

Literature survey of cell concentrations reported (in cells 100 cm^-2^) for *Amphidinium*, *Coolia*, *Gambierdiscus*, *Ostreopsis* and *Prorocentrum* for field studies that used the artificial substrate sampling method (Tester et al. 2014).

| Reference/ locations | Site Characteristics | *Amphidinium* | *Coolia* | *Gambierdiscus* | *Ostreopsis* | *Prorocentrum* | Summary of Dominant Species present |
| --- | --- | --- | --- | --- | --- | --- | --- |
| **Tan et al. 2013** |  |  |  |  |  |  |  |
| Sampadi Island, Sarawak, Malaysia Borneo |  | 5 ± 3 | 62 ± 26 | 3 ± 1 | 11± 9 | 39 ± 10 | *Coolia* > *Prorocentrum* |
| **Tester et al. 2014** |  |  |  |  |  |  |  |
| Belize | SM1 macroalgae |  |  | 96 ± 69 | 9 ± 12 | 487 ± 189 | *Prorocentrum* >*Gambierdiscus* |
|  | SM2 macroalgae |  |  | 37 ± 35 | 600 ± 451 | 684 ± 133 | *Prorocentrum* = *Ostreopsis* |
|  | SM3 macroalgae |  |  | 20,798 ± 14,086 | 160 ± 224 | 6,529 ± 1,1176 | *Gambierdiscus* >> *Prorocentrum* |
|  | SM4 macroalgae |  |  | 319 ± 127 | 554 ± 159 | 643 ± 184 | *Prorocentrum* = *Ostreopsis* |
|  | SM5 macroalgae |  |  | 33 ± 27 | 0 ± 0 | 535 ± 138 | *Prorocentrum* |
|  | SM6 macroalgae |  |  | 363 ± 127 | 6,346 ± 2,366 | 6,655 ± 2,697 | *Prorocentrum* = *Ostreopsis* |
|  | SM7 macroalgae |  |  | 431 ± 94 | 3,685 ± 384 | 2,806 ± 343 | *Ostreopsis* > *Prorocentrum* |
|  | SM8 macroalgae |  |  | 359 ± 102 | 123 ± 54 | 2,932 ± 753 | *Prorocentrum* |
|  | SM9 macroalgae |  |  | 146 ± 31 | 31 ± 12 | 635 ± 106 | *Prorocentrum* |
|  | SM10 macroalgae |  |  | 200 ± 31 | 168 ± 90 | 2,152 ± 241 | *Prorocentrum* |
|  | SM11 macroalgae |  |  | 36 ± 40 | 254 ± 143 | 1,015 ± 358 | *Prorocentrum* |
|  | SM12 macroalgae |  |  | 752 ± 62 | 451 ± 133 | 3,357 ± 510 | *Prorocentrum* |
| Malaysia | SM13 macroalgae |  |  | 8 ±8 | 80 ± 63 | 566 ± 230 | *Prorocentrum* |
|  | SM14 macroalgae |  |  | 24 ± 14 | 1,268 ± 407 | 740 ± 277 | *Ostreopsis* > *Prorocentrum* |
|  | SM15 macroalgae |  |  | 73 ± 30 | 768 ± 460 | 1,349 ± 487 | *Prorocentrum* > *Ostreopsis* |
|  | SM16 macroalgae |  |  | 26 ± 16 | 157 ± 70 | 732 ± 111 | *Prorocentrum* |
|  | SM17 macroalgae |  |  | 47 ± 25 | 137 ± 77 | 1,050 ± 681 | *Prorocentrum* |
|  | SM18 macroalgae |  |  | 22 ± 11 | 359 ± 194 | 811 ± 621 | *Prorocentrum* |
| **Yong et al. 2018** |  |  |  |  |  |  |  |
| Rawa Island, Malaysia | I. Healthy hard coral communities (> 70% cover) | 80 ± 12 | 12 ± 2 | 78 ± 14 | 2860 ± 928 | 287 ± 42 | *Ostreopsis* |
|  | II. Oral rubble with attached turf algae (70–90%) | 112 ± 22 | 13 ± 3 | 257 ± 81 | 648 ± 176 | 242 ± 35 | *Ostreopsis* |
|  | III. Sandy patches with relatively low microalgal coverage | 95 ± 13 | 13 ± 3 | 91 ± 23 | 450 ± 73 | 376 ± 46 | *Ostreopsis* *> Prorocentrum* |
|  | IV. High coverage (60%) of rubbles and fine sand | 179 ± 42 | 9 ± 3 | 101 ± 27 | 1513 ± 416 | 329 ± 50 | *Ostreopsis* |
|  | V. Extremely high abundances of macroalgae (>90%) | 39 ± 11 | 16 ± 6 | 29 ± 12 | 2186 ± 998 | 198 ± 55 | *Ostreopsis* |
| **Fernández-Zabala et al. 2019** | | | | | | | |
| The Canary Islands and Cape Verde, archipelagos of the Macaronesian region | 1 - Mixed macroalgal bed, rocks and sand |  | 2735 ± 624 | 252 ± 44 | 434,216 ± 60,263 | 1980 ± 591 | *Ostreopsis* |
|  | 2 - Mixed macroalgal bed and rocks |  | 8,329 ± 2,828 | 59 ± 102 |  | 823 ± 256 | *Coolia* |
|  | 4 - Mixed macroalgal bed and rocks |  | 8,329 ± 2,828  2,647 ± 593 | 51 ± 41 (rep 2) | 1088 ± 397 (rep 1)  19,218 ± 1,698 (rep 2) | 157 ± 116 (rep 1)  344 ± 211 (rep 2) | *Coolia* |
|  | 5 - Mixed macroalgal bed, rocks and sand |  | 676 ± 183 | 67 ± 864 | 434,313 ± 81,925 | 1176 ± 297 | *Ostreopsis* |
|  | 6 - Mixed macroalgal bed, rocks and sand |  | 50,653 ± 8,408 | 59 ± 0 |  | 98 ± 68 | *Coolia* |
|  | 7 - Mixed macroalgal bed, rocks and sand |  | 402 ± 350 (rep 1)  487 ± 121 (rep 2) | 42,192 ± 9,711 (rep 1)  445 ± 258 (rep 2) | 235 ± 61 (rep 1)  40,175 ± 4,208 (rep 2) | 971 ± 608 (rep 1)  1782 ± 393 (rep 2) | *Gambierdiscus* (rep 1)  *Ostreopsis* (rep 2) |
|  | 8 - Mixed macroalgae and coral bed, rocks and sand |  | 5,126 ± 1,133 | ± | 27,765 ± 3,571 | 2,681 ± 494 | *Ostreopsis* |
|  | 9 - *Dictyota* and coral bed, rocks and sand |  | 289 ± 133 | ± | 247,759 ± 70,409 | 1,518 ± 557 | *Ostreopsis* |
| **Mangialajo et al. 2017** | | | | | | | |
| Near Nice Côte d’Azur airport, France | artificial riprap |  |  |  | ~4-600 |  | ND |
| Villefranche-sur-mer Bay, Rochambeau, France | Mixed artificial-natural zones |  |  |  | ~1-20 |  | ND |
| Villefranche-sur-mer Bay, Grasseuil, France | Mixed artificial-natural zones |  |  |  | ~0.5-6 |  | ND |

References:

Fernández-Zabala, J., Tuya, F., Amorim, A. & Soler Onís, E. Benthic dinoflagellates: Testing the reliability of the artificial substrate method in the Macaronesian region. Harmful Algae **87**, 10.1016/j.hal.2019.101634 (2019).

Mangialajo, L. et al. Benthic Dinoflagellate Integrator (BEDI): A new method for the quantification of Benthic Harmful Algal Blooms. Harmful Algae **64**, 1-10 (2017).

Tan, T.H., Lim, P.T., Mujahid, A., Usup, G. & Leaw, C.P. Benthic harmful dinoflagellate assemblages in a fringing reef of Sampadi Island, Sarawak, Malaysia. Marine Research in Indonesia, **38**, 77-87 (2015).

Tester, P.A. et al. Sampling harmful benthic dinoflagellates: Comparison of artificial and natural substrate methods. Harmful Algae **39**, 8-25 (2014).

Yong, H.L et al. Habitat complexity affects benthic harmful dinoflagellate assemblages in the fringing reef of Rawa Island, Malaysia. Harmful Algae **78**, 56-86 (2018).

**Supplementary Data 5:**

**Summary of the results of the canonical correspondence analysis (CCA; Fig. 5).**

| Axes | 1 | 2 | 3 | 4 |  |
| --- | --- | --- | --- | --- | --- |
| Eigenvalue | 0.1088 | 0.0333 | 0.0089 | 0.0034 |  |
| Cumulative percentage variance of species explained by environmental variables | 70.5 | 92.0 | 97.8 | 100 |  |
| Correlations with environmental variables | | | | |  |
| light | 0.58255 | 0.15564 | 0.04438 | -0.56141 | *** |
| temperature | -0.66364 | 0.59117 | 0.19526 | -0.18782 | *** |
| Turf | 0.49155 | 0.36589 | 0.30378 | 0.38296 | *** |
| Fleshy macrophytes | 0.02331 | 0.45044 | -0.01119 | 0.03839 | *** |
| Hard corals | 0.15747 | -0.20881 | -0.15501 | -0.24836 | ** |
| Rubble | -0.30706 | -0.29969 | 0.65339 | -0.37470 | ** |
| Sand | -0.44064 | -0.2212 | -0.26179 | 0.28230 |  |
| Microbial mat | -0.01473 | -0.09281 | -0.506 | -0.29241 | • |
| Invertebrates | -0.02722 | -0.23578 | -0.12973 | -0.02783 |  |
| Soft corals | -0.10857 | -0.21362 | 0.33724 | 0.1012 |  |
| Sponges | -0.03375 | -0.24649 | 0.39607 | 0.28502 |  |

Significant codes: ‘***’ 0.001; ‘**’ 0.01; ‘*’ 0.05; ‘•’ 0.1

Permutation test (*n* = 999) for CCA under reduced model.

**Supplementary Data 6:**

**Coefficient of variations (CV) of benthic harmful dinoflagellate abundances determined from each microhabitat type.**

| Microhabitat | *Gambierdiscus* | *Ostreopsis* | *Coolia* | *Prorocentrum* | *Amphidinium* |
| --- | --- | --- | --- | --- | --- |
| 1 | n.a. | 1.55 | 1.22 | 2.57 | 1.32 |
| 2 | 4.69 | 2.76 | 1.10 | 0.45 | 2.50 |
| 3 | n.a. | 1.51 | 0.68 | 0.71 | n.a. |
| 4 | 2.42 | 1.69 | 1.21 | 0.82 | 1.72 |
| 5 | 1.89 | 1.27 | 0.93 | 0.80 | 1.47 |
| 6 | 1.43 | 1.64 | 1.03 | 0.89 | 0.93 |
| 7 | 2.66 | 2.30 | 1.15 | 0.85 | 2.04 |
| 8 | 3.89 | 1.63 | 1.68 | 1.70 | 1.33 |

n.a., data not available due to no cells found in the microhabitat.
